# USP28 protects development of inflammation in mouse intestine by regulating STAT5 phosphorylation and IL22 production in T lymphocytes

**DOI:** 10.3389/fimmu.2024.1401949

**Published:** 2024-07-15

**Authors:** Gwenaëlle Le Menn, Keela Pikkarainen, Daniela Mennerich, Dominika Miroszewska, Thomas Kietzmann, Zhi Chen

**Affiliations:** ^1^ Faculty of Biochemistry and Molecular Medicine, University of Oulu, Oulu, Finland; ^2^ Intercollegiate Faculty of Biotechnology of University of Gdańsk and Medical University of Gdańsk, University of Gdańsk, Gdańsk, Poland

**Keywords:** DSS-induced colitis, T cell, USP28, inflammation, IL22, Stat5

## Abstract

**Introduction:**

Ubiquitin-specific proteases (USPs), a large subset of more than 50 deubiquitinase proteins, have recently emerged as promising targets in cancer. However, their role in immune cell regulation, particularly in T cell activation, differentiation, and effector functions, remains largely unexplored.

**Methods:**

We utilized a USP28 knockout mouse line to study the effect of USP28 on T cell activation and function, and its role in intestinal inflammation using the dextran sulfate sodium (DSS)-induced colitis model and a series of in vitro assays.

**Results:**

Our results show that USP28 exerts protective effects in acute intestinal inflammation. Mechanistically, USP28 knockout mice (USP28-/-) exhibited an increase in total T cells mainly due to an increased CD8+ T cell content. Additionally, USP28 deficiency resulted in early defects in T cell activation and functional changes. Specifically, we observed a reduced expression of IL17 and an increase in inducible regulatory T (iTreg) suppressive functions. Importantly, activated T cells lacking USP28 showed increased STAT5 phosphorylation. Consistent with these findings, these mice exhibited increased susceptibility to acute DSS-induced intestinal inflammation, accompanied by elevated IL22 cytokine levels.

**Conclusions:**

Our findings demonstrate that USP28 is essential for T cell functionality and protects mice from acute DSS-induced colitis by regulating STAT5 signaling and IL22 production. As a T cell regulator, USP28 plays a crucial role in immune responses and intestinal health.

## 1 Introduction

The balance between T helper cell 17 (Th17) and T regulatory (Treg) cells is crucial in the development of multiple inflammatory diseases such as auto-immune disease, obesity, and some cancers (1–3). A better understanding of the different molecular mechanisms involved in T cell development, differentiation and/or function is essential to control this balance and develop novel strategies for the treatment of these inflammatory diseases.

Post-translational modifications (PTMs) play a crucial role in regulating the balance between Th17 and Treg cells (4–6). Among these PTMs, ubiquitination has been demonstrated to directly target FOXP3 and RORγt, which are key transcription factors involved in Treg and Th17 cell differentiation. In Treg cells, polyubiquitination of the transcription factor FOXP3, leads to its degradation through the proteasome as well as impairs Treg suppressive function (7, 8). In Th17 cells, ubiquitination of RORγt can have opposite effects depending on the E3 ubiquitin ligase utilized. RORγt ubiquitination through the E3 ubiquitin ligase TRAF5 stabilizes RORγt protein and enhances IL17 expression (9) while the E3 ligase Itch leads to RORγt proteasomal degradation and a reduction in Th17 differentiation (10).

Ubiquitination is reversible through the action of deubiquitinating enzymes (DUBs). The largest subfamily of DUBs are the ubiquitin-specific proteases (USPs), which have recently been discovered as drug targets for cancer treatment. Interestingly, FOXP3 and RORγt are not only targeted by different E3 ligases but also by multiple USPs. For example, USP44 and USP7 appeared to promote FOXP3 function in regulating Th17- and Treg-cell differentiation (11, 12). By contrast, USP4 and USP15 were found to promote Th17 immune cell differentiation through deubiquitination of RORγt (13, 14).

Apart from FOXP3 and RORγt, several other transcription factors such as STAT3, IRF4, BATF, HIF-1α, MYC, and NFAT play a role in Th17 cell differentiation (15–19), but their connections to the ubiquitin-proteasome system and the USPs involved are not well characterized. Notably, evidence from diverse cancer models supports the notion that MYC, HIF-1α, and STAT3 can be regulated by USP28 (20–22). Known for its impact on apoptosis, DNA damage, and cell proliferation, USP28 is extensively studied in cancer where it accelerates the progression and correlates with poor prognosis in various cancers, such as glioma, colorectal, and breast cancers (23). Apart from its role in cancer, the functions of USP28 beyond this context remain largely unexplored. Given that MYC is essential for the global metabolic reorganization that occurs early in activated T cells, that HIF-1α promotes Th17 differentiation under hypoxic conditions, and that STAT3 has a crucial role in Th17 cell differentiation through its association with RORγt expression, we hypothesize that USP28 may play a role in T cell development and function. To investigate this hypothesis, we utilized previously generated USP28 knockout (USP28^-/-^) and littermate control (USP28^+/+^) mice. Our study extends to explore the impact of USP28 on intestinal inflammation using acute and chronic DSS-induced colitis *in vivo* models.

## 2 Materials and methods

### 2.1 DSS colitis models

Control (USP28^+/+^) and USP28 knockout (USP28^-/-^) mice were obtained as described previously (24). All animals were maintained at the Laboratory Animal Center of the University of Oulu. All experimental procedures were performed in accordance with the license number ESAVI/7374/2019, approved by the National Project Authorization Board of Finland.

DSS was added in the drinking water of 10-12 weeks old USP28^+/+^ and USP28^-/-^ male mice to induce colitis. For the acute DSS colitis model, mice were treated with 2% DSS-water for seven days. DSS-water was replaced by autoclaved water for the following 3 days. Mice were sacrificed on Day 10. For the chronic DSS colitis model, mice received 3 cycles alternating 7 days of 1.5% DSS-water and 14 days of autoclaved water. Blood, spleen, mesenteric lymph nodes (mLN) and colon were collected on the day of the sacrifice for cytokines detection, flow cytometry, and histological staining, respectively.

### 2.2 T cell preparation and isolation

CD4+ and CD8+ cells were isolated from mouse spleen and lymph nodes respectively using L3T4 microbeads and CD8a (Ly-2) microbeads (Miltenyi). Naïve CD4+ T cells were obtained by CD4+ enrichment (CD4+ T cell isolation kit, Miltenyi) followed by positive isolation of naïve cells (CD62L microbeads, Miltenyi). All T cell isolations were performed according to the manufacturer´s instructions.

### 2.3 *In vitro* mouse cell culture

Complete RPMI1640 medium containing 10% fetal bovine serum (FBS), 2 mM glutamine, penicillin (100 IU/ml), streptomycin (0.1 mg/ml; Sigma-Aldrich), and 2.5 μM β-mercaptoethanol was used in *in vitro* cultures expect when mentioned otherwise.

Naïve CD4+ T cells were used for activation, proliferation, and polarization assays.

#### 2.3.1 T cell activation assay

T cells were activated with different concentrations: 0, 0.5, 1, 2, and 5 μg/ml, of plate-bound anti-CD3 (16-0031-86) and anti-CD28 (16-0281-85, both from eBioscience) each for 24 and 48 h, respectively. The cells were then stained with antibodies against CD69 (11-0691-85) and CD25 (12-0251-83, both from eBioscience) and were analyzed by flow cytometry.

#### 2.3.2 T cell proliferation assay

T cells were labeled with 2.5 µM CellTrace Violet dye (Invitrogen) according to the manufacturer´s instructions, then activated and cultured in the presence of anti-CD3 (1 μg/ml) and anti-CD28 (1 μg/ml) for 3, 4 or 5 days. T-cell proliferation was analyzed by flow cytometry.

#### 2.3.3 Treg cell polarization

T cells were cultured with anti-CD3 and anti-CD28 (1 μg/ml) in the presence of recombinant IL2 (10 ng/ml; R&D Systems) and TGFβ1 (10 ng/ml; PeproTech).

Th17 cell polarization: T cells were cultured with anti-CD3 and anti-CD28 (1 μg/ml) in the presence of recombinant mouse IL6 (60 ng/ml), human TGFβ1 (5 ng/ml);, human IL23 (30 ng/ml) and anti-mouse anti-IFNγ (500-P119, 1 µg/ml, all from PeproTech) in complete IMDM culture medium.

#### 2.3.4 Treg suppression assay

Celltrace violet labeled wild-type CD4+ cells (Tresp; 0.05x10^6^ cells per well) were co-cultured with polarized Treg cells in 96-well plates (1µg/ml anti-CD3/CD28). Cells were mixed at a Treg/Tresp cell ratio of 2:1 to 1:4 (serial dilution of Treg cells, factor 2). Dye dilution was analyzed by flow cytometry on day 5.

#### 2.3.5 Tc1 cell polarization

CD8+ T cells were cultured with anti-CD3 (1 μg/ml) and anti-CD28 (1 μg/ml) in the presence of recombinant mouse IL12 (20 ng/ml), and mouse anti-IL4 (500-P54, 1 μg/ml, both from PeproTech).

### 2.4 Flow cytometry

For surface staining, cells were stained in PBS + 0.5% BSA with the following antibodies: CD3 (560590), CD4 (550954), CD8 (553035), CD44 (561860), CD62L (561919), CD11b (553311), Gr1 (553129), B220 (553089), NK1.1 (553164, all from BD Biosciences, Franklin Lakes, NJ), CD69, CD25, and CD11c (11-0114-82, eBioscience, San Diego, CA) for 40 min at 4°C.

For intracellular staining of cytokines, cells were stimulated in the presence of phorbol 12-myristate 13-acetate (PMA) and ionomycin plus Golgi inhibitor for 4 hours before permeabilization and fixation steps. Foxp3 staining kit (eBioscience) and the following antibodies were used: anti-IL17A (17-7177-81), anti-IFNγ (53-7311-82), anti-Foxp3 (12-4774-42, all from eBioscience) and anti-T-bet (sc-21749, Santa Cruz Biotechnology). Stained cells were analyzed on an LSR Fortessa flow cytometer (BD Biosciences). Data were analyzed using FlowJo software (version 10, Tree Star, Ashland, OR).

### 2.5 RNA extraction and real-time quantitative PCR

RNA was extracted using the RNeasy kit (Qiagen) according to the manufacturer´s instructions. Total RNA was reverse transcribed using the SuperScript VILO cDNA Synthesis Kit (Invitrogen). Quantitative PCR was performed using either Luna universal qPCR Master Mix (Biolabs) for the SYBER Master Mix or PROBE FAST ABI Prism 2X qPCR Master Mix (Kapa Biosystems) for the TaqMan Master Mix. The corresponding primer sequences are listed below in **Table 1**. Data were analyzed using the *Hprt* gene (Applied Biosystems) as an endogenous control.

**Table 1:** Primer sequences.

Gene name	Forward primer sequence 5´-3´	Reverse primer sequence 5´-3´
*Hprt*	GTAATGATCAGTCAA CGGGGGAC	CCAGCAAGCTTGCAA CCTTAACCA
*Ifng*	GCCATCAGCAACAA CATAAGC	TGGGACAATCTCTTC CCCAC
*Ttx21*	GCCAGGGAACCGCT TATATG	GACGATCATCTGGGT CACATTGT
*Granzyme B*	CCATCGTCCCTAGA GCTGAG	TTGTGGAGAGGGCA AACTTC
*Perforin*	GCCTGGTACAAAAA CCTCCA	AGGGCTGTAAGGAC CGAGAT
*IL22*	GCCCTCACCGTGAC GTTTTA	CCACCATAGGAGGCC ACAAG
*IL2*	CTGCGGCATGTTCT GGATTT	TGTGTTGTCAGAGCC CTTTAG
*Tgf-β*	CGTGGAAATCAAC GCTCCAC	AGAAGTTGGCATGGT AGCCC

Primer sequences used for real-time quantitative PCR.

### 2.6 Western blot detection

Primary antibodies against mTOR (2983s), Akt (9272), MAPKAPK5 (7419s), Stat3 (9132), p-Stat3 (9145), Stat5 (9363s), p-Stat5 (9359s), Jak1 (3332), p-Jak1 (3331s), Jak2 (3229), and p-Jak2 (3771l) were purchased from Cell Signaling Technology; USP28 (HPA006778), and β-actin (A5441) were from Sigma Aldrich. Results were normalized according to β-actin expression that served as loading control.

### 2.7 Cytokine detection

Luminex technology [ProcartaPlex Mouse 11-Plex Mix and Match Panel (PPX-11-MXH6CNK); Thermofisher Scientific] was used to measure IFNγ, IL1β, IL10, IL12/IL23p40, IL17A, IL2, IL22, IL4, IL6, MIP-1α and TNFα in mice plasma samples. All quantifications were done according to the protocols provided by the manufacturer.

### 2.8 Histopathology

For colitis experiments, colons were excised, washed with PBS, sectioned and divided into four equal parts: proximal (prox), middle 1 (mid1), middle 2 (mid2) and distal segments. Colon segments were then fixed in 10% neutral buffer formalin for 24h at room temperature before paraffin embedding. Tissues cross sections of 5µm were stained with hematoxylin and eosin and histologic evaluation of colitis severity was performed.

Each colon section was analyzed and scored separately. The degree of inflammation was scored according to the Wirtz protocol. Sections were scored according to the following criteria: 0 = no evidence of inflammation, 1 = low level of inflammation, 2 = moderate level of inflammation, 3 = high level of inflammation, and 4 = maximum inflammation. The overall inflammation score was determined as the sum of the scores for the proximal, middle1, middle2, and distal segments.

### 2.9 Statistics

Statistical analyses were performed using the Prism 9.0 software (GraphPad Software, La Jolla, CA, USA). P values between groups were calculated using the student t-test. Differences were considered statistically significant at p <0.05.

## 3 Results

### 3.1 USP28 deficiency alters steady state immune cell composition

**Figure 1:**
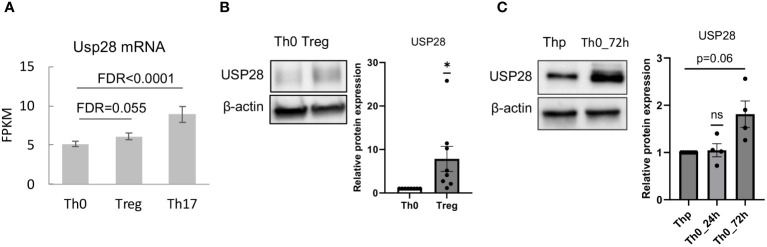
USP28 is expressed in differentiated Th17 and Treg cells. **(A)** USP28 mRNA expression level in CD4+ naïve T cells differentiated into Th0, Th17 and Treg cells analyzed by RNA sequencing (n=3). **(B)** USP28 protein expression. Representative Western blot images of one experiment (left) and USP28 protein expression relative to beta-actin (right) in *in vitro* differentiated Th0 and Treg cells (n=8). Data are expressed as mean ± SEM. One-sample t-test *p < 0.05 between control cells (Th0) and effector T cells (Treg or Th17), ns, not significant between genotypes. **(C)** Representative Western blot image of USP28 protein expression in resting naïve CD4+ cells (Thp) and activated CD4+ T cells (n=4).

Very little is known about the function of the deubiquitinating enzyme USP28 in T cell biology so far. First, we determined whether USP28 is expressed in different Th cell subsets and CD8+ cells. Analysis of RNA-seq data from our group (25) showed that USP28 was expressed in CD4+ cells and was preferentially expressed in *in vitro* differentiated Th17 compared to Th0 (control) and Treg cells (**Figure 1A**). In Treg cells, USP28 expression appeared to be increased at both the mRNA and protein levels compared to Th0 (**Figures 1A, B**). In CD8+ T cells activated under control conditions (Tc0) or differentiated under Tc1 conditions to induce IFNγ production, no difference in USP28 mRNA level was observed when comparing the two conditions (**Supplementary Figure S1A**). However, a trend towards increased USP28 protein expression was detected in CD3/CD28 activated helper T cells at 72h compared to resting naïve T cells (**Figure 1C**), suggesting that USP28 protein levels are induced by T cell activation.

**Figure 2:**
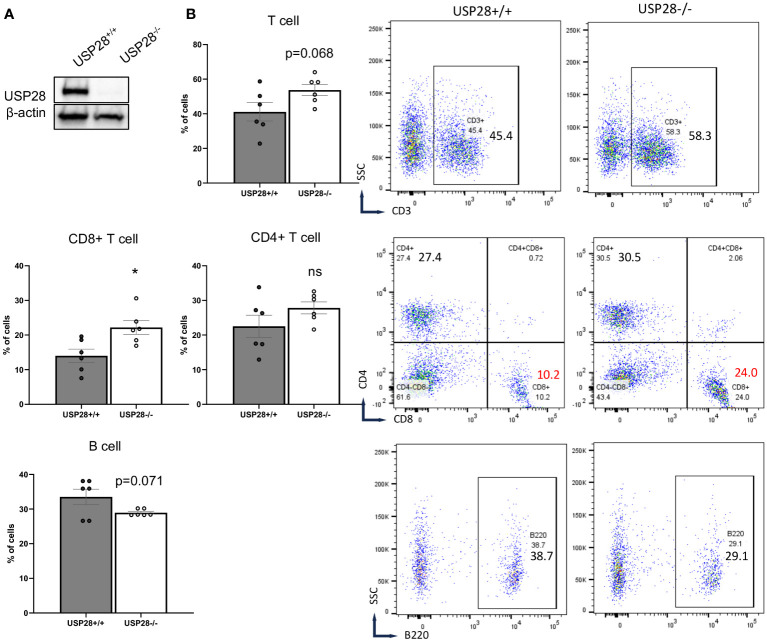
USP28 deficiency alters steady-state immune cell composition. **(A)** USP28 detection in control and USP28^-/-^ CD4+ naive cells. Representative Western blot image from three experiments. **(B)** Flow cytometry analysis of T cells (CD3+, CD8+ and CD4+ cells) and B cells in the blood of USP28^-/-^ and USP28^+/+^ mice. Data are expressed as mean ± SEM (n=5-6). Student T-test *p < 0.05 between genotypes, ns, not significant between genotypes.

To better understand the role of USP28 in T cells, USP28 knockout (USP28^-/-^) and their littermate control mice (USP28^+/+^) were generated as previously described (24). First, the knockout of USP28 in T cells was confirmed by Western blot analysis (**Figure 2A**). Characterization of immune cell populations was then performed by flow cytometry analysis in the thymus, spleen, and blood of these mice. Overall, in the thymus, although USP28^-/-^ mice exhibited a decreased proportion of DN1 cells (CD44+CD25-), no further defect of thymic T-cell development could be observed, as similar proportions of single positive CD4+ and CD8+ cells were quantified in both genotypes (**Supplementary Figure S1B**). In the spleen, the proportions of T cells (CD4+, CD8+ and, CD4+ naïve cells), NK cells and, myeloid cells are similar between USP28^-/-^ and USP28^+/+^ mice (**Supplementary Figure S1C**). Next, the lymphoid cell proportions of B and T cells appear to be altered in the blood of USP28^-/-^ mice, with a decrease in B cells and an increase in T cells compared to USP28^+/+^ mice (**Figure 2B**).

Looking at the T cells population (CD3+ cells), CD8+ cells were significantly increased while CD4+ cells (naïve and helper cell subsets such as Th1, Th17 and Treg cells) remained unchanged in the blood of USP28^-/-^ mice (**Figure 2B** and **Supplementary Figure S1C**). Similarly, the proportions of other cell types such as NK cells (NK1.1+), dendritic cells (CD11b+CD11c+), granulocytes (Gr1+), myeloid cells (CD11b+) and myeloid-derived suppressor cells (CD11b+Gr1+) were similar between USP28^-/-^ and USP28^+/+^ mice (**Supplementary Figure S1C**).

Overall, we observed a preferential expression of USP28 in Th17 and Treg cells, and an altered T cell distribution in USP28 knockout mice. These results led us to hypothesize that USP28 may play a role in T cell homeostasis or function.

### 3.2 USP28 protects mice against the early development of DSS-induced colitis

**Figure 3:**
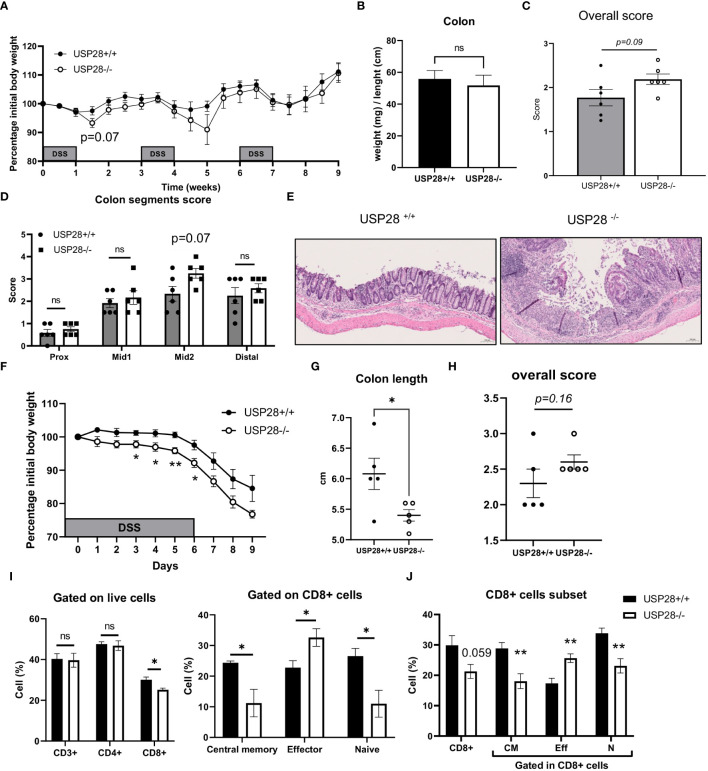
USP28 protects mice against the early development of DSS-induced colitis. USP28^-/-^ and USP28^+/+^ mice were challenged with chronic **(A–E)** or acute **(F–J)** DSS-induced colitis (n=6-7 and n=5 mice, respectively) prior to sacrifice and analysis of colitis severity and immune response. **(A, F)** Percentage of weight loss compared to the initial weight of the mice. **(B)** Colon weight/length ratio on the day of sacrifice. **(C, H)** Histology score for total colon segments and **(D)** details of histology score for each colon segment for chronic DSS-induced colitis samples. **(E)** H/E staining of mid2 colon segment. 20x **(G)**. Colon length on the day of sacrifice from acute DSS colitis. **(I)** Flow cytometric analysis of T cell subset in draining mLN and spleen **(J)** samples from acute DSS-induced colitis. Data are expressed as mean ± SEM. Unpaired T-test was used to compare between USP28^-/-^ and littermate control USP28^+/+^ mice. *p < 0.05, **p<0.01, ns, not significant between genotypes.

Since T cells play an essential role in the development of inflammation, to investigate the role of USP28 on T cell effector function, a DSS-induced colitis model was applied to USP28^-/-^ and littermate control mice. Chronic DSS-induced colitis was induced by three cycles of DSS treatment. Compared to control mice, USP28^-/-^ mice tended to lose more weight after the first two cycles of DSS feeding. However, at the end of the experiment, no differences in weight loss, spleen weight and colon weight/length ratio were observed between USP28^-/-^ and USP28^+/+^ mice (**Figures 3A, B** and **Supplementary Figure S2A**). Histological analysis was performed on the colon samples. The overall score was slightly increased in USP28^-/-^ mice compared to control mice (**Figure 3C**), and an increase in immune cell infiltration was particularly observed in the mid2 segment (**Figure 3D**). Furthermore, a disruption of mucosal structure in the mid2 segment can be observed in USP28^-/-^ DSS-challenged mice compared to USP28^+/+^ DSS-challenged mice (**Figure 3E**). Characterization of immune cells in spleen and mLN did not show any difference between the two groups (**Supplementary Figure S2B**).

Because greater weight loss was observed in USP28^-/-^ mice after the first cycle of DSS treatment, we decided to evaluate the effect of USP28 in an acute DSS-induced colitis model. Throughout the acute DSS treatment, USP28^-/-^ mice lost significantly more weight compared to USP28^+/+^ control mice (**Figure 3F**). Reduced colon length and a trend towards increased inflammatory scoring of whole colon samples were consistently observed between the two groups (**Figures 3G, H**). Flow cytometric analyses of immune cells in the mLN showed changes in the CD8+ cell subset in both spleen and mLN. USP28 deficiency resulted in a reduced proportion of CD8+ cells compared to control mice, along with a shift in the proportion of central memory, effector and naïve CD8+ cells. This shift was characterized by a decrease in memory and naive CD8+ cells in favor of effector CD8+ cells (**Figures 3I, J** and **Supplementary Figures S3A, B**). Notably, a trend toward increased frequency of IFNγ+ cells was observed in CD4- cells, possibly from CD8+ cells. No differences were observed between the two groups for B cells, NK cells, myeloid cells, T cells or CD4+ T cell subsets (**Supplementary Figures S3C, D**).

Together, USP28 protects mice against early DSS-induced colitis development and long-term intestinal structural integrity.

### 3.3 USP28 inhibits expression of IL22

**Figure 4:**
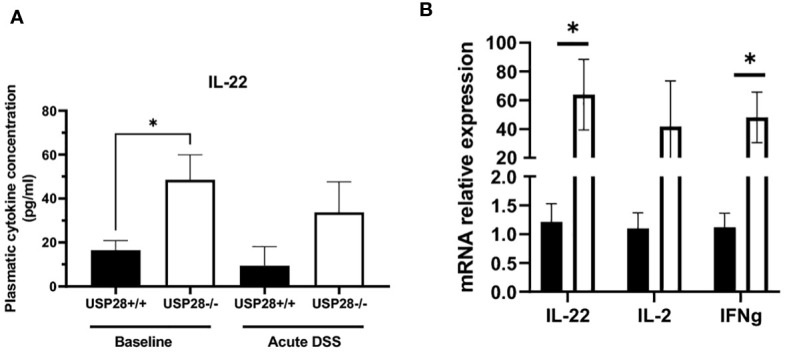
USP28 inhibits expression of IL22. **(A)** Graph showing IL22 cytokine quantification in plasma of USP28^+/+^ and USP28^-/-^ mice under baseline or acute DSS colitis condition (n=5-6). **(B)** Relative mRNA expression level of indicated genes in mLN of USP28^+/+^ and ^-/-^ mice under acute DSS colitis condition (vs. Hprt) (n=5). Data are expressed as mean ± SEM. Unpaired t-test was used to compare USP28^-/-^ and littermate control USP28^+/+^ mice *p< 0.05.

We then characterized the cytokine profile in the peripheral blood of USP28 deficient and littermate control mice from the colitis experiments. In non-challenged mice, IL22 levels stood out. They were significantly higher in USP28 deficient mice compared to control mice, whereas no significant differences in other cytokine levels were observed between the two groups. In both acute and chronic DSS-induced colitis settings, cytokine levels were comparable between USP28^-/-^ and USP28^+/+^ mice and the level of IL22 was still increased in USP28^-/-^ mice compared to control mice (**Table 2** and **Figure 4A**). To further elucidate the alterations in IL22, we conducted mRNA quantification of IL22 along with IFNγ and IL2, known to be necessary for optimal IL22 production (26) in mesenteric lymph node (mLN) samples from the acute DSS-challenged mice. The results revealed a significant upregulation in the mRNA expressions of both IL22 and IFNγ in USP28^-/-^ mice (**Figure 4B**). Together, this suggests a potential regulatory role of USP28 in modulating IL22 and IFNγ expression during acute DSS-induced colitis, with implications for the involvement of the IL2 pathway in this context.

**Table 2:** Luminex – plasma.

pg/ml	Baseline		Acute DSS		Chronic DSS	
	USP28^+/+^	USP28^-/-^	USP28^+/+^	USP28^-/-^	USP28^+/+^	USP28^-/-^
IFNg	0,0843 ± 0,08	1,46 ± 0,91	0,45 ± 0,45	2,94 ± 1,67	ND	ND
IL1b	1,46 ± 0,48	4,22 ± 3,29	2,79 ± 0,97	4,46 ± 2,93	2,31 ± 0,92	2,99 ± 2,31
IL10	ND	ND	1,62 ± 1,28	2,07 ± 1,35	2,22 ± 2,21	0
IL12	92,67 ± 14,35	103,48 ± 8,89	22,70 ± 9,34	17,99 ± 9,39	54,78 ± 12,98	77,83 ± 11,14
IL17a	8,31 ± 3,29	4,86 ± 3,30	ND	ND	ND	ND
IL22	16,49 ± 4,37	48,52 ± 11,37*	9,38 ± 8,72	33,74 ± 13,85	9,04 ± 5,72	13,1 ± 7,34
IL6	7,31 ± 6,47	3,88 ± 3,88	143,29 ± 48,75	138,19 ± 51,6	30,57 ± 17,85	19,23 ± 9,48
TNFa	0,87 ± 0,87	1,11 ± 1,11	5,71 ± 4,23	8,12 ± 4,96	ND	ND

Quantification of plasmatic cytokines in USP28^-/-^ and littermate control USP28^+/+^ mice under non-challenge (n=6), acute DSS colitis (n=5) or chronic DSS colitis (n=6) experimental conditions.

Quantification was performed using multiplex technology and data are presented as mean ± SEM, with ND indicating not determined. *p < 0.05 unpaired t-test comparing USP28^+/+^ and USP28^-/-^ mice from the same experimental condition.

### 3.4 USP28 is required for early T cell activation

**Figure 5:**
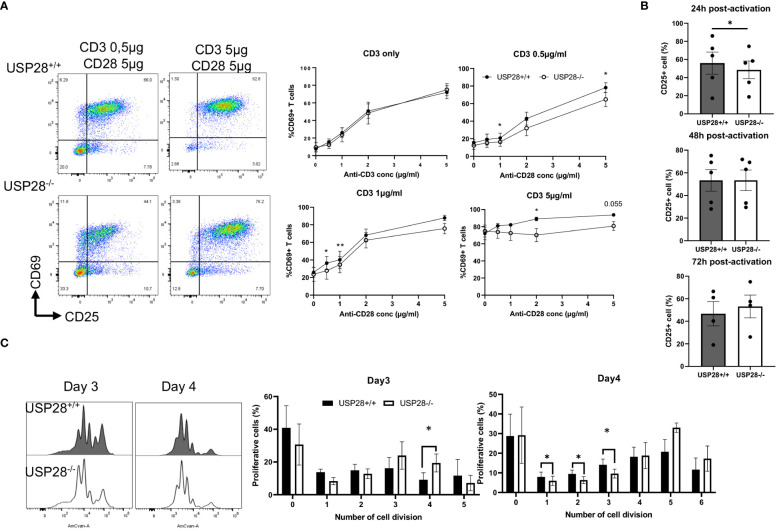
USP28 plays a role in T cell activation and proliferation through CD28/STAT5 signalling. **(A)** Flow cytometry analysis of *in vitro* naïve CD4+ T cell activation assay 24 hours after activation with increased concentrations of anti-CD3 and anti-CD28. Dot plot of one experiment and quantification of percentage of CD69+ cells (n=4). **(B)** Flow cytometric quantification of CD25 expression in T cells at 24h, 48h or 72h post activation (n=4-5). **(C)** Flow cytometry analysis of *in vitro* naïve CD4+ T cell proliferation assay after activation for 3 and 4 days. A representative histogram image of T cells labeled with Celltrace Violet (AmCyan channel) and quantification of the percentage of proliferative cells for each cell division (n=5). Data are expressed as mean ± SEM. Paired t-test was used to compare USP28^-/-^ and littermate control USP28^+/+^ T cells. *p≤ 0.05, **p<0.01.

IL22 is known to activate the JAK/STAT and MAPK pathways (27). IL2 and its downstream signaling play an important role in T cell activation and proliferation. Therefore, we decided to investigate the role of USP28 on T cell activation and proliferation *in vitro* by using USP28^-/-^ mice compared to control mice. For the T cell activation assay, naïve CD4+ cells were activated with elevated concentrations of anti-CD3 and anti-CD28 for 24h and 48h. T cell activation markers, CD69 for early activation and CD25 for mid-late activation, were then analyzed by flow cytometry (**Figure 5A**). At 24h post activation, cells only activated with anti-CD3 showed no differences in the proportion of CD69+ cells between USP28^-/-^ and USP28^+/+^ T cells. However, when the cells were activated with different concentrations of both anti-CD3 and anti-CD28, a significantly reduced frequency of CD69 expression was observed in USP28^-/-^ T cells compared to control T cells (**Figure 5A**). This effect was lost after 48h of activation (**Supplementary Figure S4A**). In response to activation, the cytokine IL2 is produced by T cells before binding to its own receptor (IL2R) on the surface of the T cells, inducing a positive feedback loop that promotes the IL2 signaling pathway (28). Therefore, the expression of CD25, the alpha subunit of the IL2R, was analyzed by flow cytometry. CD25 expression was observed in T cells at 24h, 48h and 72h after activation. Our data show that CD25 expression is significantly lower in USP28^-/-^ T cells at 24h post activation compared to USP28^+/+^ cells. The reduced CD25 and CD69 expression in USP28^-/-^ T cells were observed at an even earlier time point (**Supplementary Figure S4B**). However, with prolonged activation, CD25 expression became similar in both USP28^-/-^ and USP28^+/+^ cells (**Figure 5B**).

Next, we examined *in vitro* T cell proliferation in USP28^+/+^ and USP28^-/-^ helper T cells after 3 and 4 days of activation. The distribution of proliferative T cells for each cell division is altered in USP28^-/-^ T cells (**Figure 5C**). Our results show a decrease in the percentage of proliferative cells in the undivided and early stage of division whereas there is an increase in the percentage of proliferative cells in the late stage of division in USP28^-/-^ T cells (**Figure 5C**).

Collectively, our results suggest that USP28^-/-^ T cells have an early defect in activation that may be mediated by IL2R/CD28 signaling. However, at a later stage an increase in their proliferation rate was observed upon TCR activation compared to USP28^+/+^ control T cells. Overall, USP28 appears to alter the early phase of T cell activation and proliferation.

### 3.5 USP28 contributes to Th17 cell differentiation and iTreg cell function

We then investigated the effect of USP28 on *in vitro* T cell differentiation and effector functions. First, CD8+ cells were isolated and cultured *in vitro* under anti-CD3/CD28 activation conditions (Tc0) or Th1 like inflammatory conditions (Tc1). Flow cytometric analysis of IFNγ+ and T-bet+ cells as well as mRNA levels of these genes in USP28^+/+^ and USP28^-/-^ cells under either Tc0 or Tc1 condition did not show any differences between these two groups (**Supplementary Figures S5A, B**). However, the mRNA levels of granzyme B and perforin in Tc1 condition seemed to be increased in USP28^-/-^ CD8 cells compared to their littermate controls (**Supplementary Figure S5B**).

**Figure 6:**
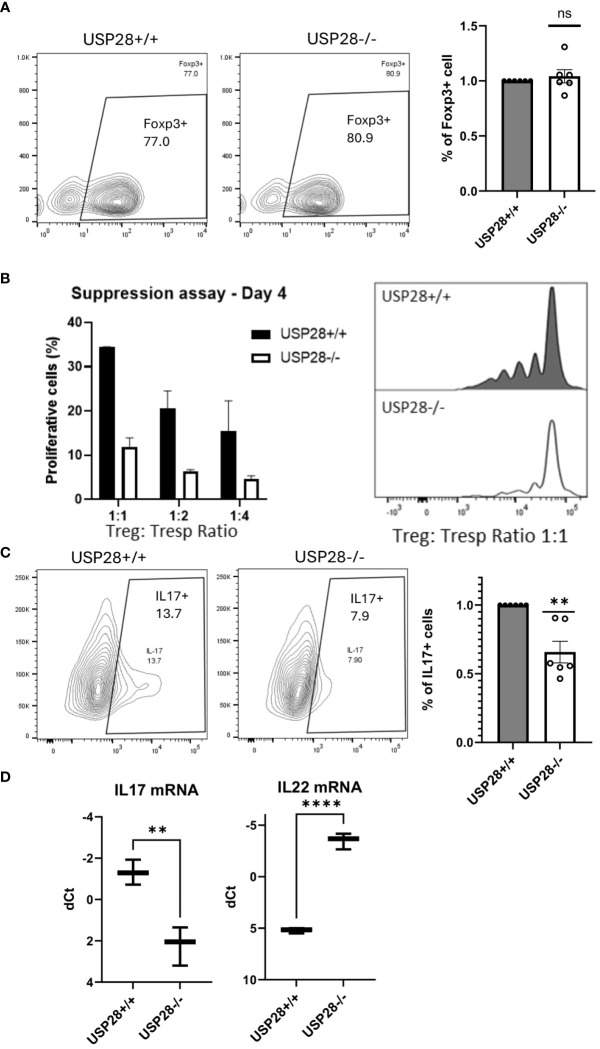
USP28 contributes to Th17 cell differentiation and iTreg cell function. *In vitro* differentiation of CD4+ naïve cells isolated from a pool of spleen and lymph node cells from USP28^-/-^ and USP28^+/+^ mice. **(A)** Flow cytometry image of a representative experiment and quantification of Foxp3+ cells in Treg (n=6) differentiated cells. **(B)** Treg suppression assay (n=2). Flow cytometric analysis of the percentage of proliferative T effector cells co-cultured with different ratios of Treg polarized cells and an image of a representative example (right panel). **(C)**. Flow cytometry image of a representative experiment and quantification of IL17+ cells in Th17 (n=6) differentiated cells. **(D)**. Relative mRNA expression level of indicated genes in Th17 polarized CD4+ cells (vs. Hprt) (n=3). Data are expressed as mean ± SEM **(A–C)**. T-test was performed to compare USP28-/- samples with littermate control. **p<0.01, ****p<0.0001 between genotypes.

Next, we investigated the role of USP28 in the differentiation of CD4+ cells into Th17 or Treg cells and analyzed the Treg suppressive function. USP28^-/-^ and USP28^+/+^ naïve CD4+ T cells were isolated and cultured in different differentiation media for 3 days before flow cytometric analysis of Foxp3 and IL17 expression in these cells. No significant differences were observed in the proportion of Foxp3+ cells in Treg differentiation between USP28^-/-^ and USP28^+/+^ cells (**Figure 6A**). Since other members of the USP family, USP7 and USP44 have been shown to be involved in Treg suppressive function (11, 12), we then performed an *in vitro* Treg suppression assay using Treg from USP28^+/+^ and USP28^-/-^ mice. The ability of Treg cells to inhibit the proliferation of responder T cells was assessed by Celltrace labelling and flow cytometry analysis. A decrease in the proliferation of responder T cells was observed when the cells were co-cultured with USP28^-/-^ Treg cells compared to control Treg cells (**Figure 6B**). On the Th17 side, we observed a consistently and significantly reduced proportion of IL17+ cells in USP28^-/-^ vs USP28^+/+^ cells under Th17 polarizing conditions at both protein and mRNA level (**Figures 6C, D**). Surprisingly, we also detected a significantly increased expression of IL22 at the mRNA level (**Figure 6D**).

Taken together, our results indicate that USP28 is not required for CD4+ Treg differentiation and Tc1 differentiation. However, USP28 is involved in Th17 cell differentiation, and more importantly, it contributes to Treg cell suppressive function.

### 3.6 USP28 regulates STAT5 signaling in T cells

**Figure 7:**
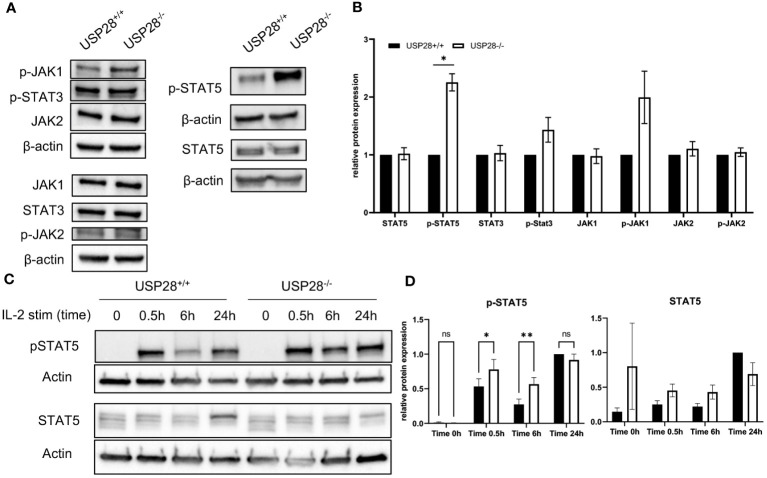
USP28 regulates STAT5 signaling in T cells. **(A, B)** Western blot analysis of the indicated proteins in USP28^-/-^ and USP28^+/+^ naïve CD4+ T cells activated *in vitro* for 3 days (n=3). **(A)** Representative Western blot images. **(B)** Relative protein quantification to β-actin. **(C, D)** Kinetics of STAT5 signaling in response to IL2 stimulation and TCR activation in USP28^-/-^ or USP28^+/+^ CD4+ cells (n=2-3). **(C)** Representative Western blot images. **(D)** Quantification of p-STAT5 and STAT5 protein relative to β-actin and calculated ratio of p-STAT5/STAT5. Data are expressed as mean ± SEM or SD. One-sample t-test in **(A, B)** and 2-way ANOVA **(D)** were used to compare between genotypes. *p < 0.05, **p < 0.01, ns, not significant.

Given our observations of decreased CD25 expression at early stages of T cell activation, but increased T cell proliferative capacity at later stages of activation, and increased IL22 expression in polarized Th17 cells, our aim was to investigate the underlying mechanisms. Given the established links between the CD28 costimulatory, IL2-activated JAK/STAT, and PI3K/AKT/mTOR pathways (28–33), we analyzed the expression of proteins related to the these pathways in activated naive CD4+ T cells (**Figure 7A** and **Supplementary Figure S6A**). The relative protein expressions of mTOR, AKT, MAPKAPK5, STAT3, p-STAT3, STAT5, JAK1, JAK2 and p-JAK2 were similar between USP28^-/-^ and control T cells, whereas we observed an increase of p-JAK1 and p-STAT5 expression in USP28^-/-^ T cells compared to control T cells (**Figure 7B**). Our results indicate that USP28 deficiency in T cells leads to an increase in the activation of JAK1/STAT5 signaling pathways. Next, we analyzed the kinetic activity of the STAT5 pathway in response to IL2 stimulation using Western blot analysis (**Figure 7C**). In response to IL2 stimulation, USP28^-/-^ T cells showed an increase in STAT5 phosphorylation after 30min and 6h, but not after 24h of stimulation compared to control T cells (**Figure 7D**). Furthermore, the increased STAT5 phosphorylation was also observed in USP28^-/-^ T cell-depleted splenocytes in response to IL7 (**Supplementary Figure S6B**). Together, these results suggest that USP28 regulates the activity of the STAT5 pathway by affecting STAT5 phosphorylation.

## 4 Discussion

In this study we have shown that USP28 plays a protective role in inflammation associated with DSS-induced colitis. We further characterized that USP28 contributes to T cell activation, subsets differentiation and/or function, possibly by mediating through the STAT5 pathway. As a member of the IL10 cytokine family, IL22 is secreted by various immune cell subsets such as Th17/Th1, CD8+ Tc22 subset cells, γδ T cells or innate lymphoid cells (ILCs) (34–37). Previous studies have shown that IL22 positively regulates epithelial homeostasis while also activating pro-inflammatory immune responses, leading to a dual role in inflammatory diseases (38, 39). Among the various immune cells analyzed, only CD8+ T cells seem to be affected by the deletion of USP28. The increase in CD8+ T cells in the blood and the increase in effector CD8+ T cells in the mLN in non-challenged mice and in mice with acute DSS colitis, respectively, were associated with elevated level of IL22 in the same organs. There is a possibility that USP28 deletion would favor CD8+ T cells producing IL22. In this case, the increased expression of granzyme B and perforin in CD8+ cells would result in a highly cytotoxic profile of these cells in USP28^-/-^ mice, which would be consistent with the exacerbated symptoms seen in acute DSS colitis. However, we cannot rule out the possibility that other immune cells are responsible for the increased IL22 production as we used constitutive USP28 knock-out mice and did not examine other immune cells.

Prior to cytokine production or T cell differentiation, T cell activation is required and is achieved through the recognition of at least two distinct signals, the TCR and the co-stimulatory CD28 molecule. During this process, inhibitory, degradative or activating ubiquitination and deubiquitination action can occur (40, 41). Some members of the USP family, such as USP18, USP12 and USP9X, are known to directly affect the CD28 signaling pathway. Depletion of USP18 leads to hyperactivation and overproduction of IL2 in T cells, whereas deficiency of USP12 and USP9X causes a decrease in NF-κB activation, which subsequently reduces proliferation and cytokine production upon TCR activation (42–44). Compared to other USPs, nothing is known about the role of USP28 in T cell activation or the potential target protein associated with this pathway. However, in our study, we observed a transient defect of the CD28/IL2R signaling pathway in USP28^-/-^ T cells upon early activation. Since STAT5 regulates the expression of CD25 (IL2RA), the increased expression of CD25 and increased T cell proliferation at a later time point may be due to increased STAT5 phosphorylation (45).

While examining proteins involved in T cell activation/proliferation signaling pathways, we observed an over-activation of the STAT5 pathway associated with an increase in STAT5 phosphorylation levels as well as a decrease in total STAT5 protein in USP28^-/-^ T cells. In non-small-cell lung cancer, USP28 appears to mediate STAT3 signaling through deubiquitination and stabilization (21). Although that study did not report changes in STAT5 levels, a similar interaction between USP28 and STAT5 would be consistent with our results in which ubiquitination of STAT5 in the absence of USP28 would lead to its proteasomal degradation (46). Our results seem to show a different role of USP28 on the STAT5 pathway.

Upon binding to its receptor, IL22 activates several signaling pathways, including phosphorylation of JAK1, TYK2 and STAT1, STAT3 and STAT5 (27, 47). STAT5 has been reported to regulate IL22 expression (26). In this study, we observed both increased STAT5 phosphorylation and enhanced IL22, suggesting that USP28 is involved in the IL22/STAT5 positive regulatory feedback loop.

Finally, STAT5 and IL2 are also known to play a central role in Treg development and function (48). Treatment with neutralizing antibodies against IL2, transgenic mice lacking IL2, IL2R or STAT5 show a deficit in Treg cells and develop autoimmune disease (49, 50). Treg suppressive function can be restored by expressing a gain-of-function form of STAT5 (51). The increased activation of the STAT5 pathway in USP28^-/-^ T cells may therefore be linked to the higher suppressive function found in the Treg cells. Importantly, STAT5 has been shown to suppress Th17 differentiation (52), here we observed the reduced IL17 expression in USP28^-/-^ T cells most likely due to increased STAT5 phosphorylation.

In conclusion, using USP28^-/-^ mice, we have uncovered the essential role of USP28 in multiple aspects of T cell functionality. Our data demonstrate that USP28 contributes to the protective effect for the early development of intestinal inflammation by regulating STAT5 signaling and IL22 production.

## Data availability statement

The original contributions presented in the study are included in the article/**Supplementary Materials**, further inquiries can be directed to the corresponding author/s.

## Ethics statement

The animal studies were approved by National Project Authorization Board of Finland. The studies were conducted in accordance with the local legislation and institutional requirements.

## Author contributions

GM: Data curation, Investigation, Visualization, Writing – original draft. KP: Investigation, Writing – review & editing. DMe: Investigation, Visualization, Writing – review & editing. DMi: Investigation, Writing – review & editing. TK: Supervision, Writing – review & editing. ZC: Conceptualization, Data curation, Funding acquisition, Investigation, Project administration, Resources, Supervision, Visualization, Writing – review & editing.
