# Supplementary figures and images for: USP28 protects development of inflammation in mouse intestine by regulating STAT5 phosphorylation and IL22 production in T lymphocytes
Source: Front Immunol. 2024 Jul 15;15:1401949. doi: 10.3389/fimmu.2024.1401949 (PMC11284026; doi:10.3389/fimmu.2024.1401949)

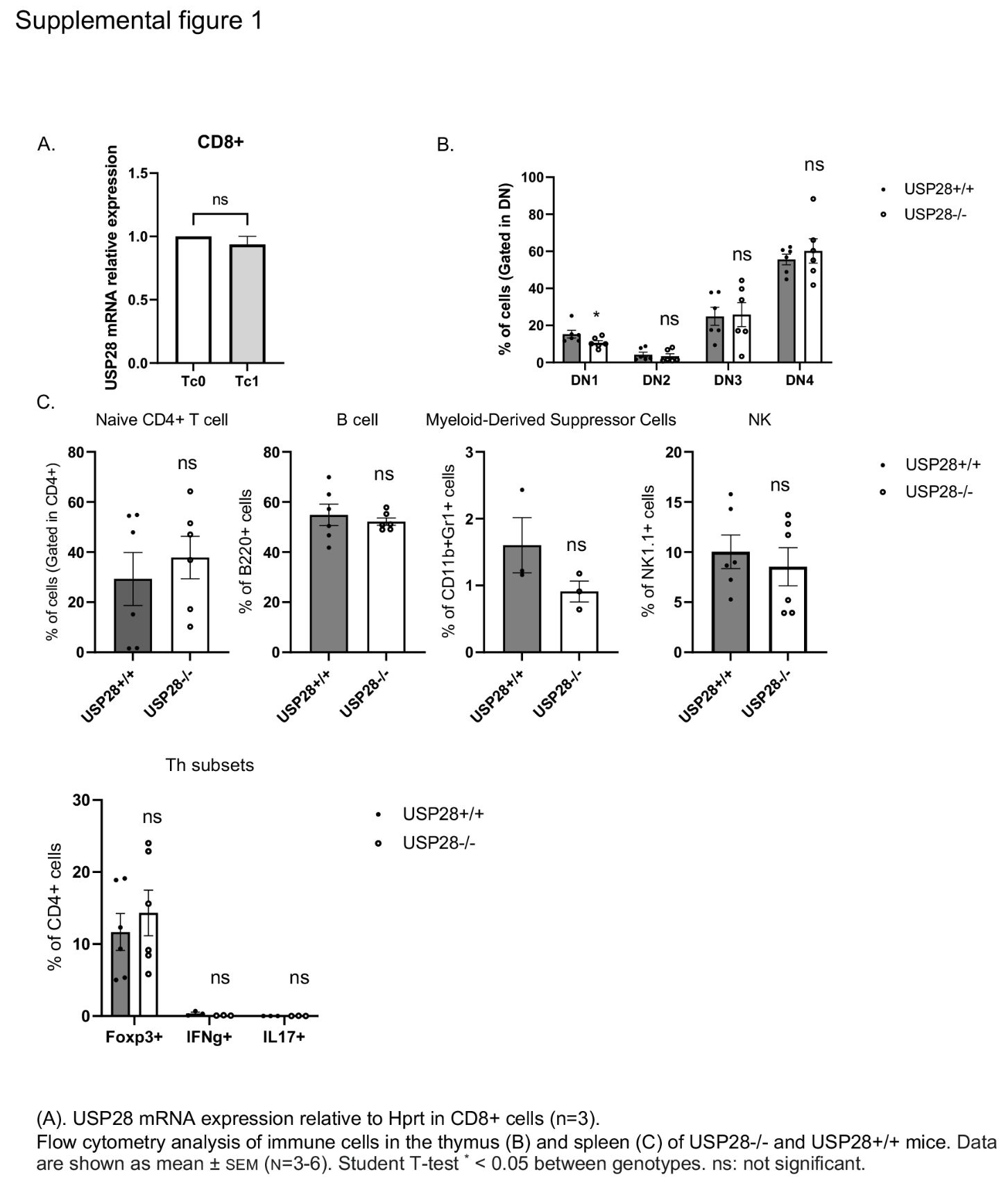


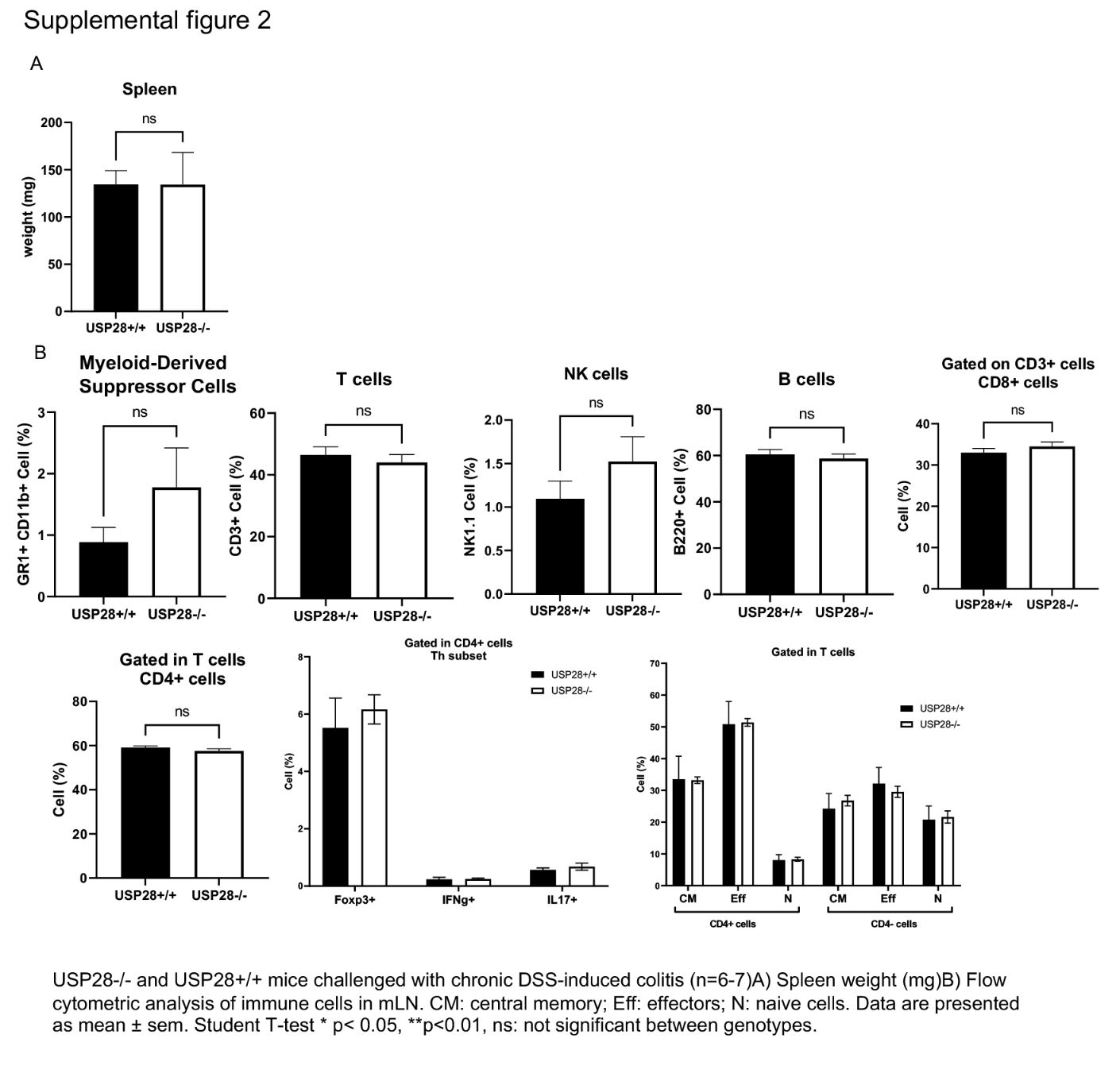


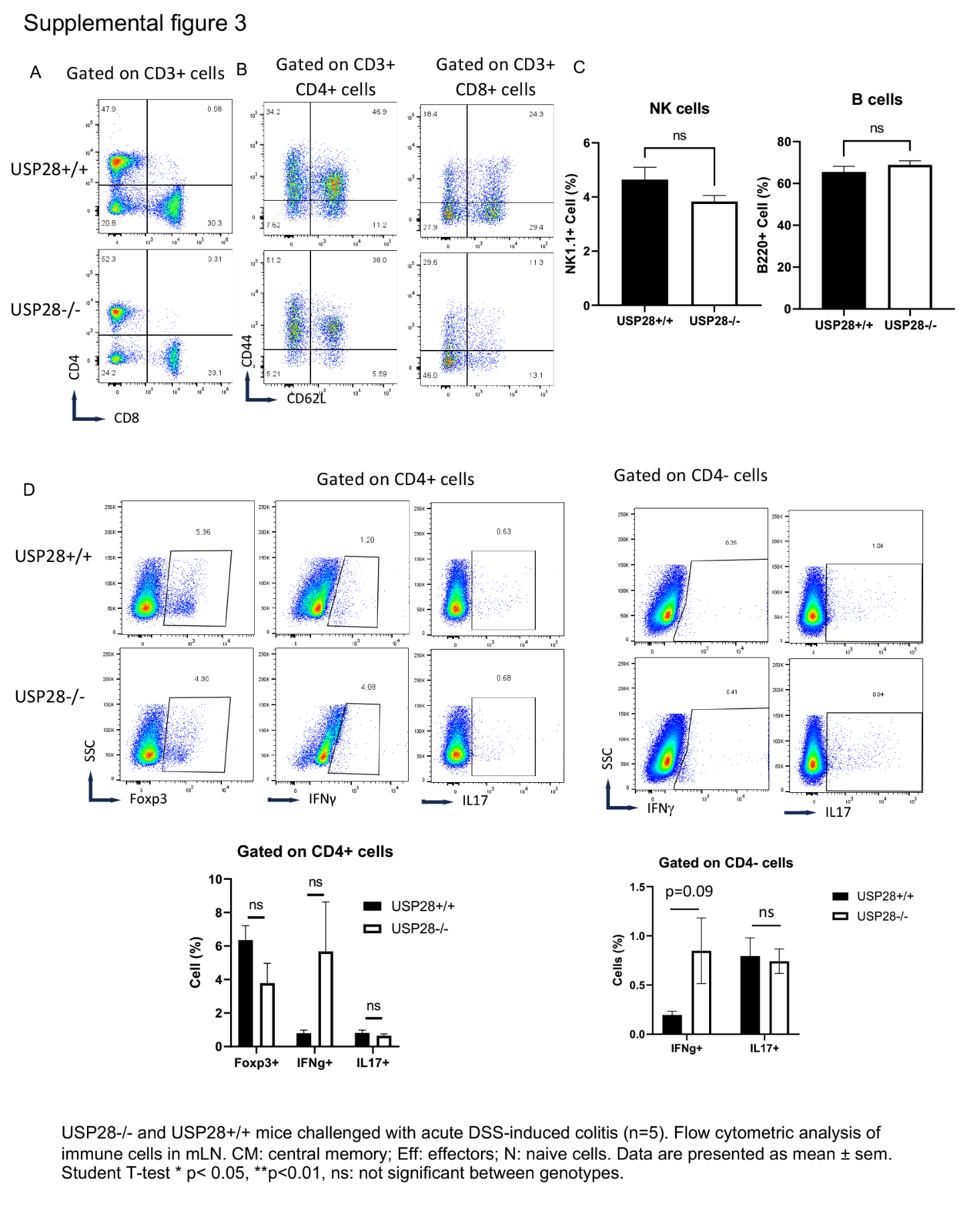


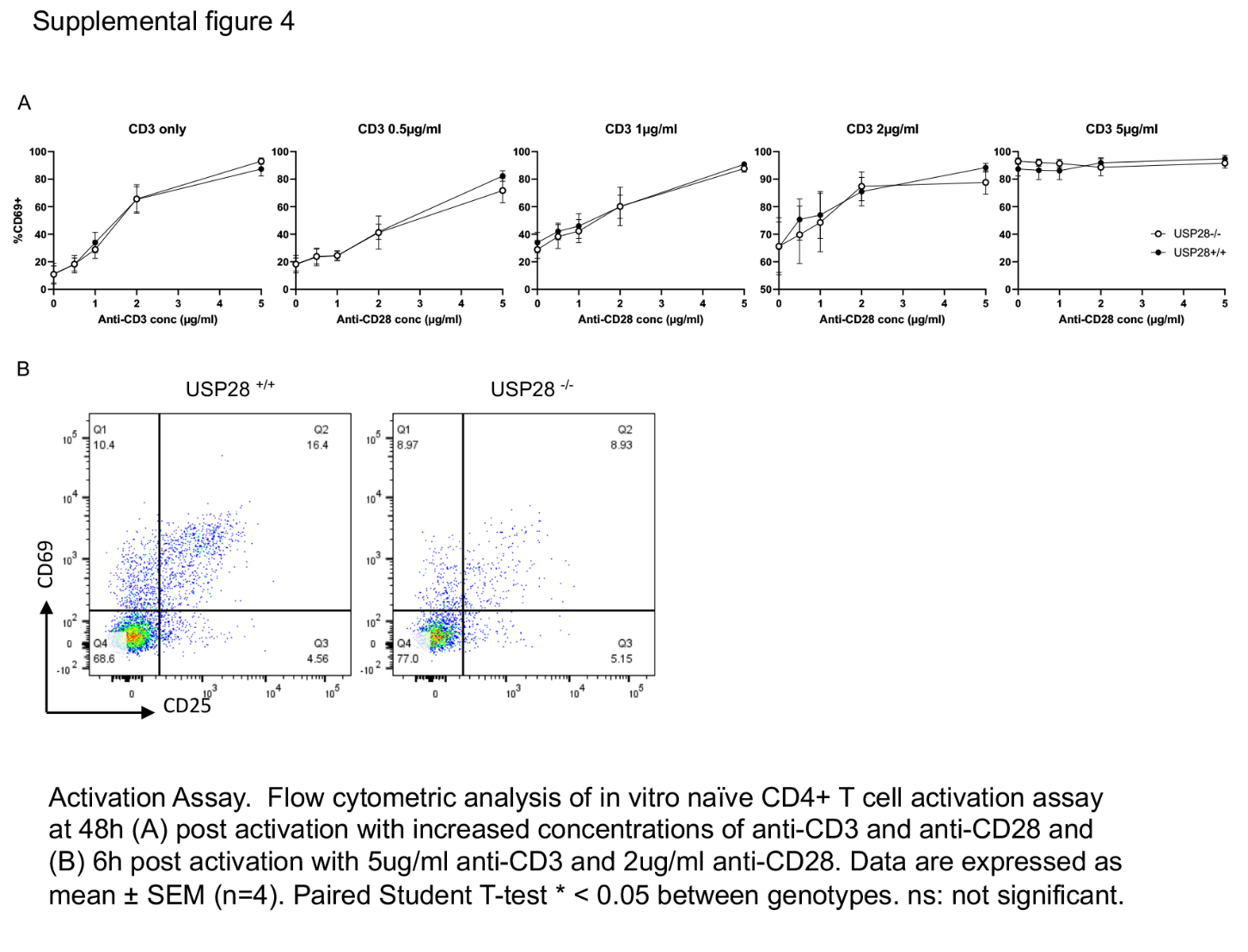


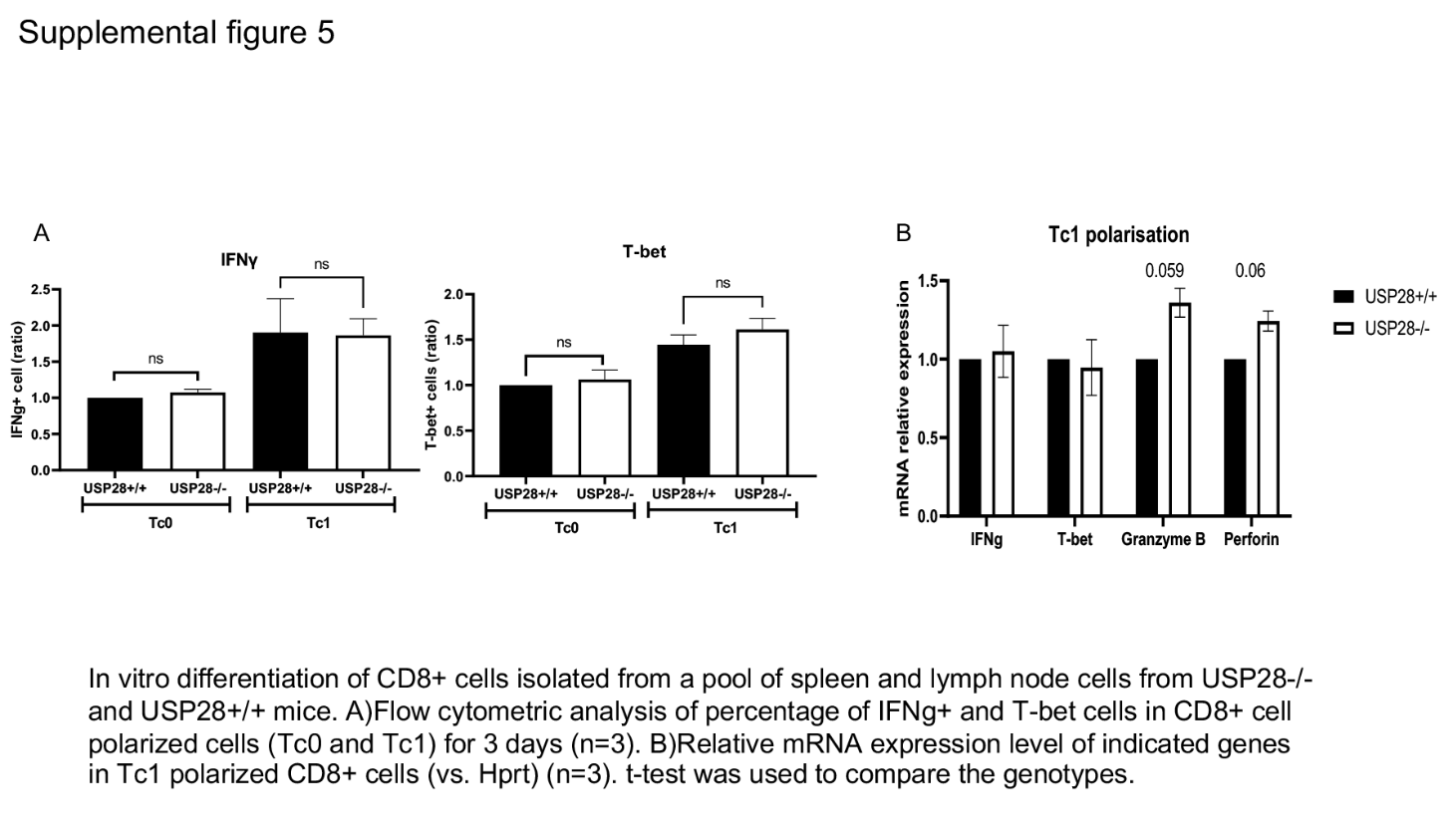


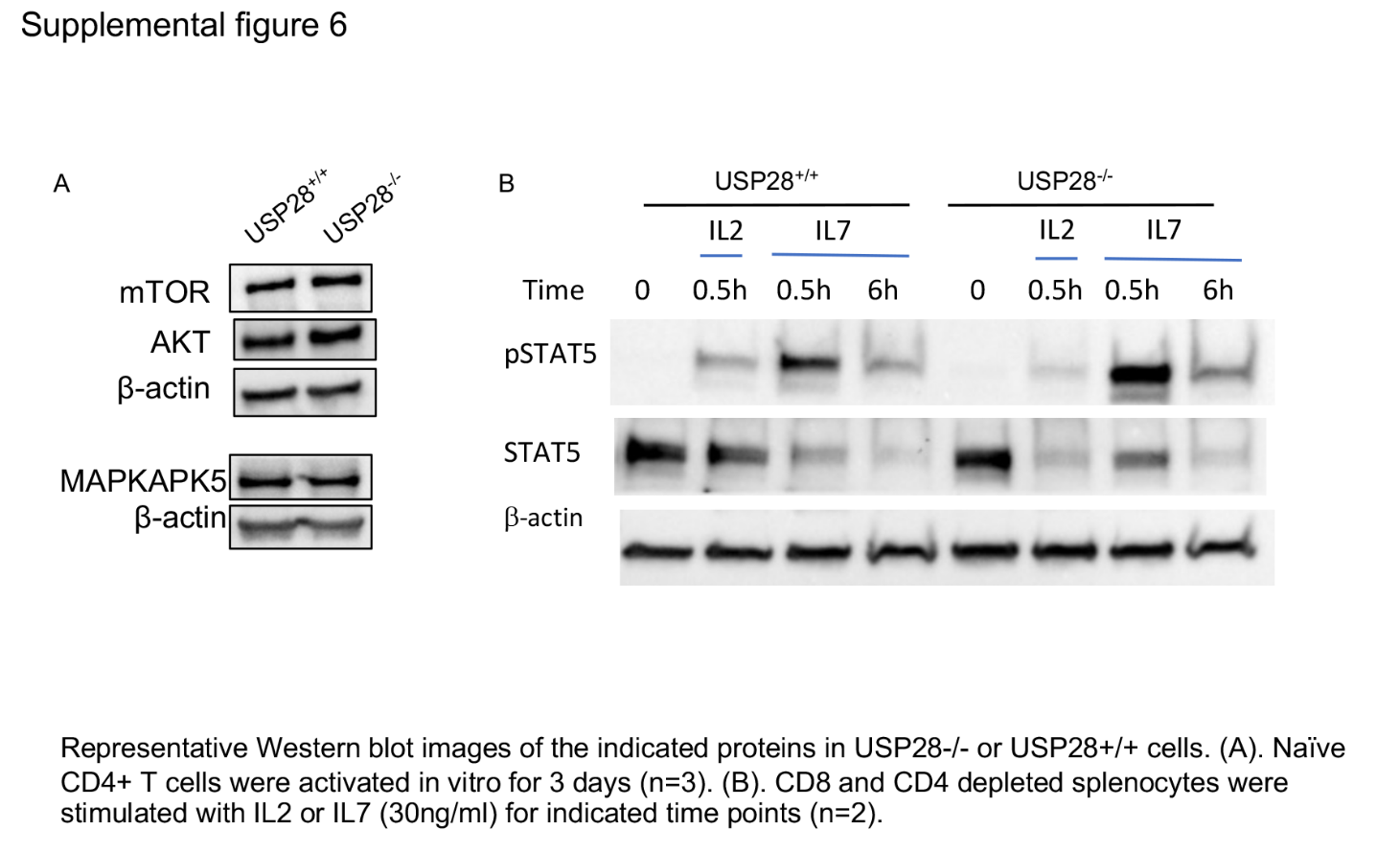

Supplement: Supplementary file 1 [file DataSheet_1.docx]
